# How suitable is freshwater sponge Ephydatia fluviatilis (Linnaeus, 1759) for time-integrated biomonitoring of microbial water quality?

**DOI:** 10.1099/acmi.0.000691.v4

**Published:** 2024-04-19

**Authors:** Allison Cartwright, James S. G. Dooley, Chris D. McGonigle, Joerg Arnscheidt

**Affiliations:** 1 Ulster University, Cromore Road, Coleraine, Northern Ireland, BT52 1SA, UK

**Keywords:** water-quality monitoring, faecal pollution, freshwater sponges, *Ephydatia fluviatilis*, *Enterococcus faecalis*, *Escherichia coli*

## Abstract

Faecal pollution of water by bacteria has a negative effect on water quality and can pose a potential health hazard. Conventional surveillance of microbial water quality relies on the analysis of low-frequency spot samples and is thus likely to miss episodic or periodic pollution. This study aimed to investigate the potential of filter-feeding sponges for time-integrated biomonitoring of microbial water quality. Laboratory trials tested the effects of different ratios of bacterial abundance and the sequence of exposure on bacterial retention by the freshwater sponge *Ephydatia fluviatilis* (Linnaeus, 1759) to establish its potential to indicate bacterial exposure. Gemmule grown sponges were simultaneously exposed to *Escherichia coli* and *Enterococcus faecalis* but at different ratios (Trial 1) or individually exposed to each bacterial species but in different sequential order (Trial 2). The * E. coli* and *E. faecalis* retained in each sponge was quantified by culture on selective agars. Data analysis was conducted using the Kruskal–Wallis test and/or the Mann–Whitney U test to compare between the numbers of bacteria retained in each treatment. Additionally, the Wilcoxon matched-paired signed-rank test was used for comparison of the different bacterial abundances retained within each individual sponge. Sponges from all trials retained *E. coli* and *E. faecalis* in small numbers relative to the exposure (<0.05 % Trial 1 and <0.07 % Trial 2) but exhibited higher retention of *E. coli*. Higher abundance of either bacterial species resulted in significantly lower (*P*<0.005) retention of the same species within sponges (Trial 1). An initial exposure to * E. coli* resulted in significantly higher (*P*=0.040) retention of both bacterial species than when sponges were exposed to *E. faecalis* first (Trial 2).Bacterial retention by sponges was neither quantitatively representative of bacterial abundance in the ambient water nor the sequence of exposure. This implies either selective filtration or an attempt by sponges to prevent infection. However, freshwater sponges may still be useful in biomonitoring as qualitative time-integrated samplers of faecal indicator bacteria as they detect different bacteria present in the water even if their quantities cannot be estimated.

## Data Summary

The raw data for the bacterial counts, sponge sizes, bacteria per mm^2^ of sponge and % of added bacteria retained are given for each treatment in each trial. Trial 1 was the bacteria retained when sponges were exposed to *E. coli* and *E. faecalis* in different ratios. Trail 2 was the bacteria retained when sponges were exposed to *E. coli* or *E. faecalis* first and the opposite second. All data used is available from https://figshare.com/s/da59abc86c8d0e7887d7.

## Introduction

Freshwater and transitional water bodies can receive high inputs of bacteria including enterococci and *Escherichia coli* from human and animal waste [1,3]. This bacterial pollution not only compromises the integrity of aquatic ecosystems but can also have negative effects on human health as sewage contains pathogenic bacteria and viruses [2,4]. Furthermore, Metcalf *et al*. [5] found that faecal indicator bacteria can bind with other anthropogenic pollutants, including glass and microplastics, allowing them to persist in the environment for over 25 days and so they may be present long after a pollution event. Recently, within the UK, there has been an increase in public awareness of storm overflow management, which allows wastewater including storm flow and raw sewage to be released directly into water systems. In 2022, from 13 080 monitored storm overflows, there has been a total of 301 091 events with the release of untreated wastewater into English water systems [6]. All these events contribute to faecal pollution of aquatic systems, and in extreme cases they cause mass fish mortality in river systems. For example, in 2017 Anglian Water (UK) released around 6 million litres of raw sewage killing 5000 fish, resulting in a fine of £560 170 [7]. Therefore, faecal indicator bacteria are monitored so that the public can be warned when the level of water contamination presents an increased risk of exposure to harmful pathogens [8,9]. Among the bacteria routinely sampled to indicate microbial water quality are *E. coli* and *Enterococcus* spp. [5,8, 9]. If counts of these, or other faecal indicator bacteria, exceed the acceptable threshold values set by regulatory agencies, the water should not be used for bathing or drinking purposes until bacterial counts drop to safe levels [10,12].

Regulators conventionally evaluate microbial parameters based on low-frequency, single-spot water samples [13,14]. This type of sampling can show bacterial abundance through culture-based quantification after membrane filtration or through tests with defined substrate technology like colilert. Yet it will only indicate the bacterial load at a few random points in time and is thus likely to miss episodic, periodic or persistent pollution. However, filter feeders, such as sponges, process waterborne bacteria and potentially also accumulate bacteria from episodic pollution over an extended period of time when post-event water samples would not detect them anymore. When sponges are feeding, they have the ability to retain bacteria in their mesohyl [15,17]. A sponge sampled at any given point will contain bacteria, which have been filtered from the water either and are awaiting digestion or have been incorporated as symbionts [10,17]. Thus, the quantification of bacteria retained within sponges could be used as an indication of the filter organisms’ exposure to waterborne bacteria. Therefore, this study aimed to investigate the potential of freshwater sponges to be used for time-integrated biomonitoring of bacteria to document any episodic faecal pollution that may occur between sampling dates of low-resolution surveillance. We determined the retention of the faecal indicator bacteria *E. coli* and *Enterococcus faecalis* by *Ephydatia fluviatilis* in a novel model system *to* quantify bacterial retention by sponges. This sponge species was selected for being the most common freshwater sponge in Ireland, its wide geographic range and its ability to retain bacteria in a laboratory mesocosm [18,19]. Previous studies had shown that the bacteria *E. coli* and *E. faecalis* did not negatively affect *E. fluviatilis* in laboratory experiments over the timescale of our experiments [19,20]. The experimental approach was to test individual sponges in laboratory microcosms regarding the effect of exposure to these bacteria in different abundance ratios and with varied sequence of exposure.

## Methods

Two laboratory trials were conducted to explore the retention of bacteria by sponges and how this related to different modes of exposure. Trial 1 tested simultaneous exposure of sponges to *E. coli* and *E. faecalis*, but at different abundance ratios. Trial 2 exposed sponges to either *E. coli* or *E. faecalis* before removal of sponges for subsequent exposure to the other bacterial species. These trials were conducted to establish if the abundance of *E. coli* and *E. faecalis* retained by sponges were a quantitative representation of their numerical ratio in the ambient water laboratory microcosms (Trial 1) or whether it responded to the sequence of exposure to different bacteria in laboratory microcosms (Trial 2).

### Culturing of sponges

*Ephydatia fluviatilis* (Linnaeus, 1759) gemmules were surface sterilized by submersion in 1 % H_2_O_2_ for 10 min prior to hatching (modified method from [21]. To allow for movement of the sponges, gemmules were hatched onto a piece of 4 cm^2^ transparency film (Xerox type 1) in a 6 cm diameter petri dish containing 10 ml UV-treated (10 min at 254 nm) mineral water. Culturing in mineral water enabled high hatching rates as it contains silica required for spicule formation. Seven-day-old sponges were used for all experiments with their individual horizontal dimensions (mm^2^) measured at the start of the trials.

### Culturing of bacteria

The bacteria used for these experiments were *E. coli* GFP (ATCC 25922GFP) and *E. faecalis* (MW01105 – [22]. Before each experimental addition of bacteria, a 10 ml overnight culture was grown of each bacteria culture separately. Each culture was produced using 10 ml of nutrient broth in a universal tube to which one bead coated with the specific bacteria was added from the −80 ℃ stock culture. This suspension was mixed and incubated at 37 ℃ for 18 h.

### Bacterial addition – (Trial 1) different ratios of *E. coli* and *E. faecalis*

Universal tubes with 18 ml of UV-treated mineral water had 2 ml of bacterial suspension added from the overnight nutrient broth culture as sponges showed an ability to filter this concentration [20]. There were three treatments with different *E. coli: E. faecalis* ratios: 10 : 90; 50 : 50; 90 : 10, and there were 22 replicates of each treatment. For each replicate a single sponge on a transparency film was placed into the tube, so that it was leaning on the wall, to minimize settling of bacteria on sponge surfaces due to deposition. In total this trial thus tested 66 sponges. Tubes were kept at 20 °C for 24 h as the optimal duration Willenz *et al*. [19] had identified for experiments aiming at maximal bacteria retention by *E. fluviatilis*. Control tubes also received the respective bacteria suspensions but no sponges.

### Bacterial addition – (Trial 2) separate exposure to *E. coli* and *E. faecalis* in different sequence

There were two treatments in this trial. One treatment tested initial exposure to *E. coli* followed by exposure to *E. faecalis*; the other tested exposure in the reverse order, i.e. initial exposure to *E. faecalis* followed by exposure to *E. coli*. Universal tubes with 19 ml of UV-treated mineral water had 1 ml bacteria suspension added (either *E. coli* or *E. faecalis*) before a single sponge was introduced on transparency film and positioned to lean on the tube’s interior side wall. The tubes were incubated at 20 °C for 24 h before the sponge was removed and washed. The sponge was then placed in a fresh universal tube containing 19 ml of mineral water and 1 ml of the suspension of the bacterial species that the sponge had not been previously exposed to. These were incubated at 20 °C for a further 24 h. Control tubes also received the respective bacteria suspensions but no sponges. Each treatment had eight replicates. However, one sponge in the treatment with initial exposure to *E. coli* died and this replicate was therefore excluded from data analysis. Thus, the trial tested a combined total of 15 individual sponges.

### Sample processing (Trials 1 and 2)

After exposure to bacteria, transparency films with sponges were removed and washed with autoclaved deionized water three times to remove bacteria from the surface before being placed in a fresh tube with 20 ml of UV-treated mineral water. These were left for a further 24 h to allow the sponges to incorporate the bacteria retained. To end the trial, sponges and transparency film were once again washed, as described above, before the sponges were lifted and placed into an Eppendorf tube with 1 ml of autoclaved water. Each Eppendorf tube was vortexed for 2 min to extract the bacteria before tenfold serial dilutions to 10^−6^. The vortex action disintegrated the sponge into single cells so any live bacteria could be quantified. The samples for bacteria analysis were tested for *E. coli* and *E. faecalis* using MacConkey No. 3 and Slanetz and Bartley agars, respectively. The dilutions were plated onto each selective medium in six 20 µl aliquots and incubated at 37 °C for 48 h before total viable colonies were counted. After mixing water and bacteria concentrations were quantified as c.f.u. ml^−1^ with serial dilution.

### Data visualization and analysis

For comparison, counts were converted to c.f.u. per mm^2^ of sponge surface for each individual sponge as follows:

$$B_{s}=\frac{N}{A}$$

where *B*_S_=bacteria per 1 mm^2^ of sponge tissue, *N*=bacteria extracted from the sponge into 1 ml of water, *A*=individual sponge area (mm^2^)

The bacterial numbers in all sponge replicates were used to calculate the arithmetic means and standard error values for each experimental group, for *E. coli* or *E. faecalis*. The bacterial counts from sponges for each bacteria type was converted to represent the percentage retained of the original *E. coli* and *E. faecalis* added to the experimental tube. Statistical tests were carried out in SPSS (RRID:SCR_002865) using the bacterial counts from each sponge. Kolmogorov–Smirnov tests showed that bacteria retention was not normally distributed, and so different treatments were tested for significant differences (*P*<0.05) with Kruskal–Wallis tests and post-hoc pairwise comparisons using Bonferroni corrected Mann–Whitney U tests (Trial 1) or the Mann–Whitney U test (Trial 2). To test for significant differences (*P*<0.05) in the bacteria retained by the individual sponges, Wilcoxon matched-paired signed-rank tests were conducted for both trials.

## Results

The laboratory experiments quantified the sponges’ response to the simultaneous exposure with *E. coli* and *E. faecalis* at different ratios (Trial 1) and to a different sequential order of exposure with an identical abundance of the two bacterial species (Trial 2). In Trial 1 all the sponges survived and in Trial 2 all except one sponge survived, yielding a total survival rate of 98.8 % for the 82 sponges in both trials combined. Table 1 compiles the concentration ranges of waterborne bacteria at experiment start and after 24 h exposure (experiment end) in treatments and controls. Concentrations of culturable bacteria dropped by factor 10^3^ in the controls and factor 10^4^ in treatments with sponges.

**Table 1.** Concentration ranges of culturable waterborne bacteria at start and end of the experiments with 24 h exposure

	c.f.u. ml^−1^ at the start		c.f.u. ml^−1^ in controls at the expt end		c.f.u. ml^−1^ in treatments at the expt end	
	Minimum	Maximum	Minimum	Maximum	Minimum	Maximum
*E. coli*	2.3×10^7^	3.8×10^8^	4.0×10^4^	6.5×10^5^	3.4×10^3^	1.4×10^4^
*E. faecalis*	3.0×10^7^	3.0×10^8^	1.2×10^4^	4.6×10^6^	1.4×10^3^	1.5×10^4^

### Retention of *E. coli* and *E. faecalis* in sponges exposed to different ratios of both bacteria (Trial 1)

**Fig. 1.**
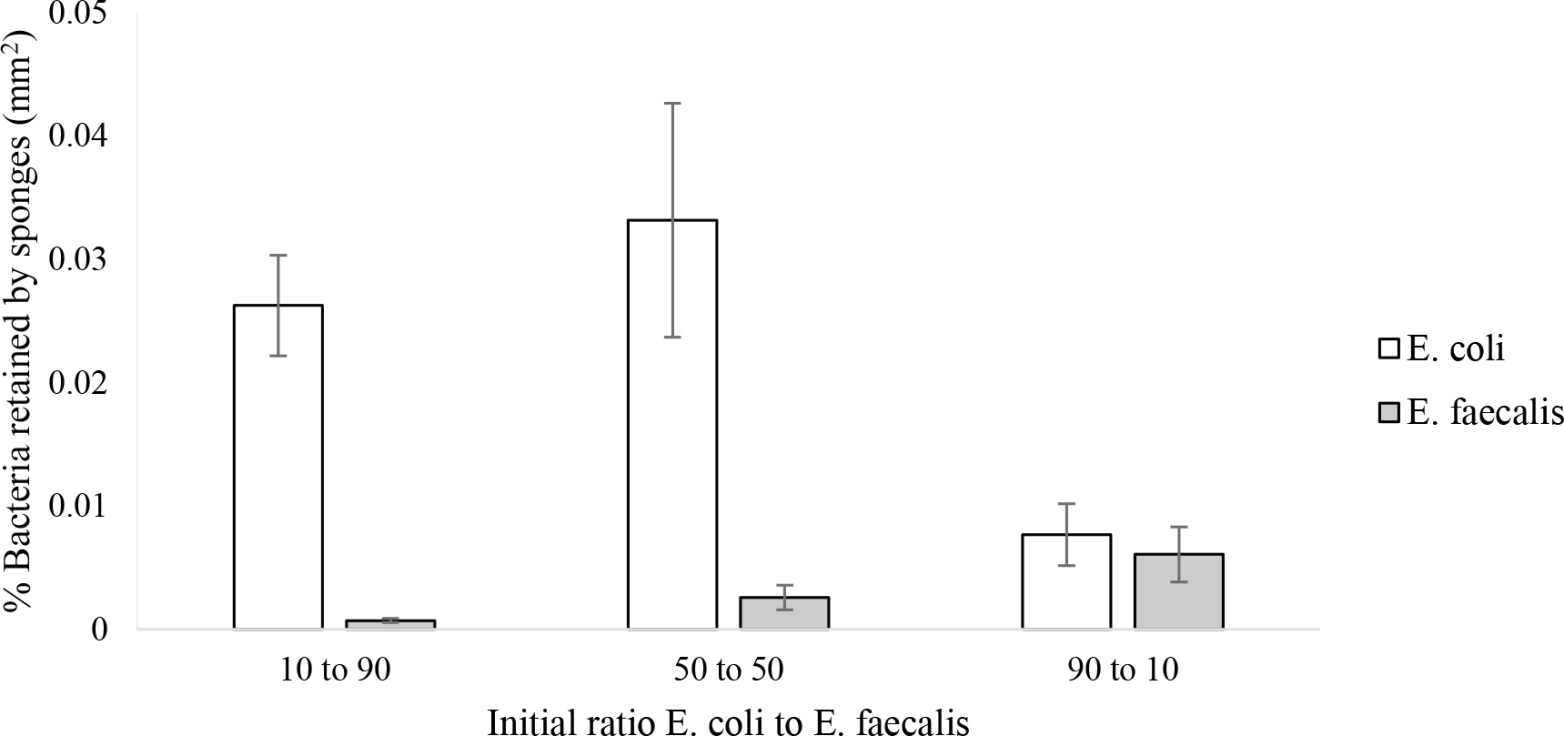
Arithmetic means and standard error values for the retention percentage of *E. coli* and *E. faecalis* by sponges in treatments with different *E. coli: E. faecalis* ratios. Different letters indicate significant differences (*P*<0.05) between bars e.g. bars with letter ‘a’ are significantly different from all other letters but not from other bars with ‘a’.

The percentage of bacterial retention by sponges was very low regardless of the initial bacterial concentration, and the bacterial retention of sponges never exceeded 0.05 % (Fig. 1). Considering each bacterial species separately (*E. coli* or *E. faecalis*), there was a significant difference between the bacterial retention for the different ratios of exposure (*E. coli* H=14.25, *df*=2, *P*=0.001; *E. faecalis* H=13.24, *df*=2, *P*=0.001). The *E. coli* retention by sponges was significantly higher when it constituted 10 and 50 % of the bacterial suspension than when its relative abundance was 90 %(10-90 % U=95 *P*<0.001; 50 %−90 % U=136, *P*=0.005). A similar pattern was observed for *E. faecalis* retention whereby the amount retained was higher at 10 % than 50 % and was significantly lower when it represented 90 % of the assemblage of waterborne bacteria (10 %–90 % U=405 *P*=0.001; 50–90 % U=136, *P*=0.005). Thus, higher relative abundances of both tested bacteria appeared to result in lower retention within the sponge.

Considering the combined pattern of both bacterial species, retention values for *E. coli* were always higher than for *E. faecalis* in individual sponges, even when *E. coli* formed only 10 % of the bacterial exposure. When sponges were exposed to 10 % *E. coli* and 90 % *E. faecalis* or assemblages with 50 % representation of both bacteria, they retained significantly higher abundances of *E. coli* (10 % *E. coli*: 90 % *E. faecalis*- Z<0.001, *P*<0.001; 50 % *E. coli:* 50 % *E. faecalis*- Z<0.001, *P*<0.001). However, when sponges were exposed to a ratio of 90 % *E. coli* and 10 % *E. faecalis*, this difference was not significant (Z=112, *P*=0.429). Therefore, high abundances of *E. coli* inhibited the sponges’ ability to retain either bacterial species.

### Retention of *E. coli* and *E. faecalis* in sponges after sequential exposure to both bacteria species (Trial 2)

**Fig. 2.**
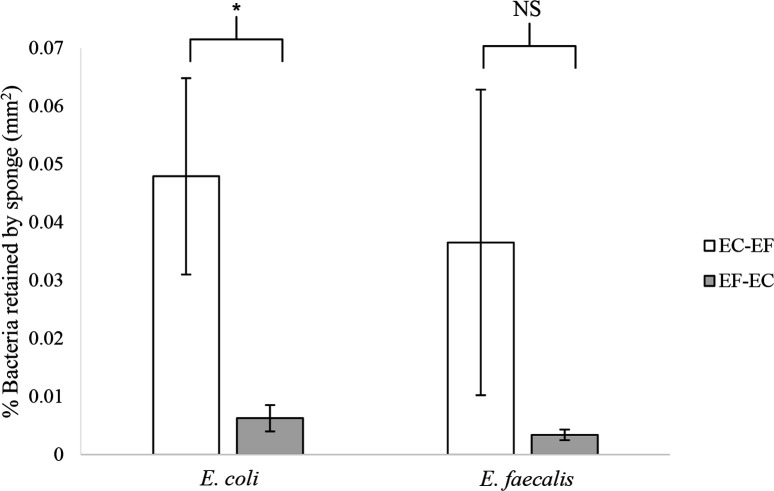
Arithmetic means and standard error values for the retention percentage of *E. coli* and *E. faecalis* by sponges in treatments with sequential exposure to bacteria (EC-EF=first exposure to *E. coli*, then to *E. faecalis*; EF-EC=first exposure to *E. faecalis*, then to *E. coli*). * – significant (*P*<0.05), NS – not significant.

The percentage of bacterial retention by sponges was very low regardless of the sequence of exposure; bacteria retained in sponges never exceeded 0.07 %. The sponges showed higher retention of *E. coli* regardless of the sequence of exposure (Fig. 2). However, the amount of *E. coli* retained in sponges was significantly higher when sponges were exposed to this bacterial type first (U=11, *P*=0.040). While the mean quantity of *E. faecalis* retained in sponges was lower when they were exposed to these bacteria first, the observed differences were not significant (U=20, *P*=0.397). Regardless of the exposure sequence, individual sponges retained similar levels of each specific bacteria (*E. coli* before *E. faecalis*- Z=8, *P*=0.161; *E. faecalis* before *E. coli*- Z=12, *P*=0.735). Interestingly, sponges first exposed to *E. coli* and then *E. faecalis* retained higher levels of both bacterial species than in sponges with an exposure in the reverse order. Overall, *E. fluviatilis* retained bacteria from all phases of the sequential exposure, but there was no consistent bias in bacterial abundance towards the most recent exposure.

## Discussion

Our study showed the ability of *Ephydatia fluviatilis* to retain bacteria from ambient water. It has been widely demonstrated that other sponge species can retain and even concentrate waterborne bacteria within their tissue [16,23, 24]. Stabili *et al*. [24], for example, recorded significantly higher bacteria numbers in sponges than the surrounding water with faecal coliforms at the abundance of 4.6 MPN g^−1^ from the marine sponge *Hymeniacidon perelevis* under fish farms while these were 0.01 MPN g^−1^ in water. The efficacy of sponges to filter water and retain waterborne bacteria can deplete the water zone of bacteria immediately above sponge colonies [25]. However, in the present study *E. fluviatilis* only retained a very low abundance of experimentally added bacteria and in the tested bacterial abundance range this was not reflective of sequence or intensity of exposure. The level of bacterial survival in this study’s controls confirmed previous research documenting 260 d survival of *E. coli* in autoclaved river water without substantial decline [26]. Lleò *et al*. [27] provided similar evidence for *E. faecalis* in excess of 12 days. Contrary to studies in river water our 24 h experiments were conducted in mineral water, whose lack of organic substrates supporting bacterial metabolism explains the observed decline of c.f.u. in controls, because a proportion of bacteria would be expected to enter a viable but non-culturable state in such conditions. Potential causes for sponges not reflecting their bacterial exposure regarding sequence and intensity include the still comparatively high experimental bacteria concentrations in our experiments, the use of juvenile sponges, sponge selectivity in filtering of bacteria, a potential infection of sponges or the activation of an immune response.

At the start of the trials very high doses of bacteria were added to the tubes which were several orders of magnitude above the concentrations normally expected in aquatic systems, for example, Daniels [28] determined an abundance range from 1.9×10^3^–2.8 x 10^4^ c.f.u. per 100 ml for *E. coli* and 3.3×10^3^–7.9 x 10^5^ c.f.u. per 100 ml for *E. faecalis* in rural Irish low-order streams 24 h after manure application during rainfall. Furthermore, Campos *et al*. [29] found concentrations of *E. coli* ranging from 1.8×10^4^–9.6×10^4^ c.f.u. ml^−1^ in raw sewage. The initial loading of bacteria in this study albeit higher than a natural level was intended to simulate a pollution event while investigating a bacterial exposure level comparable to that of studies which had quantified filtration or retention of bacteria by sponges; e.g. Francis and Poirrier [30] and Willenz *et al*. [19] conducted experiments exposing sponges to *E. coli* concentrations of 1×10^8^ to 2.20×10^9^ c.f.u. ml^−1^, respectively. Willenz *et al*. [19] showed that bacteria concentrations of this order of magnitude do not compromise the filtration system of *E. fluviatilis* and its ability to remove micro-organisms from ambient water reducing the concentrations by 3.7×10^9^ to 5.1×10^7^; however, the authors did not provide the data of bacterial decline in the water of controls. With the same validated filtration test design for gemmule grown juvenile sponges our *E. fluviatilis* colonies actually achieved a greater reduction of waterborne *E. coli* concentrations in 96 h filtration tests reducing aquatic bacteria from 1.4×10^8^ to 8.7×10^6^ [31]. With an obvious dependence on colony size, higher clearance rates and bacteria accumulation have been reported for large colonies of marine sponges [10,32]. Yet our study was conducted with juvenile sponges grown from gemmules. Unlike established sponge colonies, the gemmules from which juvenile colonies are grown can be surface sterilized to exclude any associated bacteria other than those added during the experiments.

Recent evidence for selective feeding in sponges has documented that some species may indeed be able to filter specific strains or groups of bacteria at a faster rate [33,34] and there is evidence that differences in digestibility could encourage selective feeding [33]. In accordance with the aforementioned studies, our study showed that *E. fluviatilis* retained more *E. coli* than *E. faecalis* regardless of the level of experimental exposure to these bacteria. However, others have found some marine sponges appear to have no selectivity in their retention ability [17,35]. Our study appears to be the first to provide information on the rates of the filtration or retention by sponges for *Enterococcus* and bacteria assemblages, which include *Enterococcus*, while most previous research has been conducted on *E. coli*. As Gram-positive and Gram-negative bacteria these test organisms differ in their cell-wall structure and this may also lead to different digestibility.

The observed low retention of bacteria could also result from an infection of the sponge tissue, which may compromise a colony’s filtration ability and digestive system. Sponges can succumb to bacterial infection where their cells become overrun with bacteria causing death to the organism [36]. Fu *et al*. [37] demonstrated the infection of the marine sponge *Hymeniacidon perleve* with *Vibrio* spp. through genetic markers for cell death. However, *E. coli* did not infect the sponge species they investigated. Many studies have successfully used *E. coli* for the investigation of sponge filtration rates [19,32, 38] and our own filtration tests [31] do not suggest that exposure to these bacteria had a negative effect on *E. fluviatilis* in 24 h. While there appears to be no information on the pathogenicity of *E. faecalis* for sponges, it has been demonstrated by fluorescent *in situ* hybridization that *E. faecalis* cells attach and aggregate on the external and internal structure of gemmule grown *E. fluviatilis* [20], which may indicate an infection capacity. Biofilms of enterococci have been found to release surface aggregation proteins by quorum-sensing regulators that can trigger a virulence mode thus causing infection [39,40]. If *E. faecalis* can infect sponges, there could be an evolutionary advantage in ejecting or egesting these bacteria selectively and this may indeed explain their low retention by sponges throughout our study. Such a response would compromise the suitability of sponges for quantitative time-integrated monitoring.

When exposed to only one of the two bacteria species in these trials before subsequent exposure to the other species, *E. fluviatilis* retained more *E. coli* regardless of the sequential order of exposure. This confirms that sponges can be qualitative indicators of bacterial presence for an extended time period; however, the abundance of retained bacteria in a sponge also does not appear indicative of the sequence of exposure to different bacteria. Yet this does not prohibit applications of sponges for sampling a historic pollution signature. For example, if a faecal pollution episode occurred 2 days before sampling, a sustained presence of faecal indicator bacteria would be unlikely in a water sample while a sponge sample could allow for its detection through the presence of *E. coli* or enterococci as two groups of indicator organisms likely to be released by any sewage pollution event and used as indicators for different jurisdictions. Future studies should therefore investigate the length of time sponges can retain filtered bacteria post-exposure and the minimal concentration and load of bacteria required in exposure to be detectable in a sponge sample. This would test the suitability of sponges for time-integrated qualitative surveillance monitoring of water quality.

## Conclusion

This study presents evidence that sponges may be suitable for the detection of microbes associated with current and historic faecal pollution episodes. Investigated juvenile *Ephydatia fluviatilis* colonies retained a lower proportion of waterborne bacteria especially when these had been in very high abundance thus demonstrating that sponge tissue analysis will only yield qualitative but not quantitative data for the presence of aquatic faecal pollution for biomonitoring purposes. This study also presents first evidence for retention of *E. coli* over *E. faecalis* by freshwater sponge *E. fluviatilis*. Sponges can thus provide a time-integrated qualitative record of the presence of indicator bacteria from episodic faecal pollution in aquatic systems. They may therefore be suitable for surveillance monitoring of water quality where only low-frequency sampling is feasible and episodic pollution events are likely. Monitoring of natural bathing water sites may be a potential application as conventional monitoring relies on water sampling intervals of 1 week or longer. Future studies should therefore validate detection limits, i.e. the minimal bacterial exposure required for retention by sponges and the duration of detectable retention as evidence of past pollution events. These would help to determine the suitability of sponges for qualitative time-integrated monitoring of faecal indicator bacteria in aquatic systems, especially if the required minimal bacteria concentrations for retention by sponges were aligned to health relevant water-quality thresholds.

## Abbreviations

c.f.u.: colony forming unit
MPN: most probable number
UV: ultraviolet light
